# Exploring caregivers’ experiences when seeking mental health care services for their children with autism spectrum disorder: A qualitative study from South Africa

**DOI:** 10.4102/sajpsychiatry.v32i0.2661

**Published:** 2026-07-03

**Authors:** Bathokozile M. Sithole, Keabetswe Mogase, Tshepiso D. Moeketsi

**Affiliations:** 1 Department of Psychiatry, Faculty of Health Sciences, University of Pretoria, Pretoria, South Africa

**Keywords:** autism spectrum disorder, children, caregivers, access to mental health care, South Africa, qualitative research, barriers to care

## Abstract

**Background:**

The increasing prevalence of autism spectrum disorder (ASD) highlights the importance of understanding caregivers’ experiences in accessing mental healthcare for their children, particularly given the demanding nature of supporting children with ASD in resource-constrained settings.

**Aim:**

This study aimed to explore the experiences of caregivers of children with ASD when accessing mental healthcare services in South Africa.

**Setting:**

This qualitative study was conducted at the Child and Adolescent Psychiatric Unit at Weskoppies Hospital, a tertiary-level hospital in Pretoria West, Gauteng province, South Africa.

**Methods:**

This qualitative case study purposively sampled 15 caregivers of children with ASD at a tertiary psychiatric facility in South Africa. Data were gathered through in-depth interviews and analysed using a Grounded Theory-informed thematic analysis approach.

**Results:**

Five main themes emerged: (1) challenges in accessing mental health care services, (2) delays in screening and early diagnosis, (3) limited ongoing education and knowledge-sharing, (4) the need for emotional support and counselling, and (5) facilitating referrals to specialised schools and allied health care services.

**Conclusion:**

The study identified significant barriers to mental health service utilisation, underscoring the urgent need for improved service accessibility, caregiver support and increased public awareness.

**Contribution:**

The findings advocate for strengthened mental healthcare access and targeted caregiver support for families of children with ASD in South Africa.

## Introduction

Mental healthcare is a critical component in the management of children with autism spectrum disorder (ASD). Interests about the availability, accessibility and effectiveness of these services are growing for researchers, parents and communities, particularly in low- and middle-income countries such as South Africa (SA).^1,2^ These interests are heightened by the South African constitutional obligation to provide accessible quality healthcare for all.^3^ Access to mental healthcare services is also essential for preserving the legal rights of children with ASD and their families and enabling early diagnosis, effective intervention and improved developmental outcomes.^4^

Autism spectrum disorder is a lifelong neurodevelopmental condition influenced by genetic and environmental factors.^5^ It is characterised by difficulties in emotional regulation, social interaction, communication, repetitive behaviours and restricted interests as described in the Diagnostic and Statistical Manual of Mental Disorders.^5^ The global prevalence of ASD is estimated at approximately 1 in 54 children, although rates vary significantly across studies and regions.^6^

As the global prevalence of ASD continues to rise, access to appropriate and affordable care presents a growing challenge.^7^ In low-resource settings, barriers to care remain widespread despite increasing research attention.^8^ These barriers may be related to various reasons, including access to mental healthcare services, inadequate healthcare resources and insufficient specialised expertise among providers.^8^ Wallace-Watkin et al. reported that families identified limited awareness, a shortage of trained professionals, prolonged waiting times and financial constraints as significant impediments to accessing mental healthcare for children with ASD.^9^ Furthermore, Malik-Soni et al. found that clinicians frequently lack the specialised knowledge required to effectively screen, diagnose and refer individuals with ASD.^8^

In the South African context, political history is a critical factor contributing to barriers in accessing healthcare. The enduring legacy of apartheid has entrenched disparities that continue to influence access to mental health services. These disparities are evident in the country’s bifurcated healthcare system, comprising a government-funded public sector serving the majority and a private sector accessible mainly to those with higher socio-economic status. The public healthcare sector faces challenges such as overburdened facilities, extended waiting times and a shortage of specialists, which collectively restrict service accessibility.^10^ In contrast, the private healthcare sector offers faster and more specialised care.^11^ This inequity disproportionately affects children with ASD, whose families often incur substantial personal costs to obtain necessary medical treatment and resources.^2^ Such an imbalance contravenes the United Nations’ Sustainable Development Goal 3, which advocates for universal health coverage that ensures equitable access to quality care without financial burden.^12^

Currently, the South African health system follows a tiered referral pathway.^13,14^ The pathway begins with primary-level services delivered by nurses, social workers and community health workers (CHWs); progresses to secondary-level care involving multidisciplinary teams; then tertiary academic hospitals with subspecialists such as child and adolescent psychiatrists, psychologists, social workers and occupational therapists; and culminates with quaternary care.^15^ Even when primary care is available, its lack of therapeutic interventions and access to specialised professionals, such as speech and occupational therapists, is important in the treatment of ASD.^16^ The disparities can be further appreciated in children from rural and underserved communities who face additional barriers to healthcare services, through long travel distances, poor public transportation and limited awareness of available resources.^1^ In response, the SA government has introduced the National Health Insurance (NHI) bill to mitigate some of these disparities.^7^

This study explored the lived experiences of caregivers seeking mental health services for their children with ASD.

## Research methods and design

This study employed a qualitative case study design, conducted within a single tertiary psychiatric facility. Data were analysed using a Grounded Theory-informed thematic analysis approach, which involved constant comparison of data across participants and iterative memorandum writing to track emerging analytical insights.

### Setting

This qualitative study was conducted at the Child and Adolescent Psychiatric Unit at Weskoppies Hospital, a tertiary-level hospital located Pretoria West, Gauteng province, South Africa. The unit is a specialised subspecialty service that provides inpatient and outpatient mental healthcare for children and adolescents, as well as support for their families.

### Study population and sampling

Fifteen caregivers of children with ASD were purposively sampled. Eligible patients were identified through existing files of children diagnosed with ASD and attending the Child and Adolescent Psychiatry unit at the study site, after which their accompanying caregivers were approached for participation. In-depth interviews were conducted using open-ended questions to explore participant experiences. Thematic analysis was employed to identify recurrent patterns and themes in the data.

### Inclusion criteria

The following participants were eligible for inclusion:

- Caregivers of children with ASD, with or without a comorbid psychiatric diagnosis, who were admitted to the child and adolescent unit.
- Caregivers of children with ASD, with or without a comorbid psychiatric diagnosis, attending the child and adolescent outpatient clinic at Weskoppies Hospital.
- Caregivers who are 18 years or older.
- Caregivers who were willing to participate in the study.
- Caregivers who were fluent in any of the South African official languages.

### Exclusion criteria

The following participants were eligible for exclusion:

- Caregivers who declined participation in the study.
- Caregivers who were unable to provide informed consent.
- Caregivers who represented a duplicate caregiver for the same child (in cases where both parents were available, only one was included to prevent redundancy of experiences and data duplication).

### Data collection and analysis

All participants provided written informed consent prior to data collection, and confidentiality and ethical guidelines were strictly adhered to throughout the study. Interviews were conducted by the principal researcher in English, Setswana, IsiXhosa or IsiZulu, each lasting approximately 45–60 min. A predeveloped set of open-ended questions guided each conversation while allowing participants flexibility to elaborate on their personal experiences. Interviews were audio-recorded and supplemented with field notes. Data saturation was reached after nine interviews, as no new codes or themes emerged during analysis. Data collection continued to 15 participants to confirm saturation and ensure analytical rigour.

All interviews were transcribed verbatim. Those conducted in Setswana, IsiXhosa or IsiZulu were first transcribed in the original language and then translated into English by a professional transcriber. The principal investigator, fluent in all three languages, independently verified each translation against the original audio recordings to ensure linguistic accuracy and contextual fidelity. For quality control, the co-authors randomly compared audio recordings and the transcripts.

Analysis followed a Grounded Theory approach involving constant comparison across transcripts. Initial open coding was conducted through colour-coded categorisation of participant quotations, with each colour representing a discrete code. Related codes were subsequently grouped through axial coding to identify patterns and generate overarching themes. Coding was performed by the principal investigator, and all themes were reviewed and confirmed through consensus discussion with the co-authors.

### Reflexivity and trustworthiness

The principal investigator was a mental healthcare practitioner working within the South African public health system. This professional positioning not only informed sensitivity to participant experiences but also carried potential for interpretive bias. To mitigate this, open-ended questions were used to allow participants to lead the narrative, reflexive memos were maintained throughout the research process, and themes were reviewed through consensus discussions with the research co-authors.

### Ethical considerations

The study was approved by the University of Pretoria’s Faculty of Health Sciences Master of Medicine Committee and the University of Pretoria Ethics Committee (Ethical Clearance Reference Number: 564/2023), dated 25 October 2023. All participants received written information about the study prior to enrolment. Written informed consent was obtained from each participant, including consent to audio-record interviews. Participation was entirely voluntary, and participants were informed of their right to withdraw at any time without consequence. No harm was inflicted on participants. Confidentiality was maintained throughout by assigning unique participant identifiers in place of personal information. Data are securely archived at the Department of Psychiatry, Weskoppies Hospital and will be retained for 15 years from the date of publication in accordance with institutional policy.

## Results

Five major themes, each with corresponding subthemes, emerged from the transcribed data (Table 1). These themes reflected the shared experiences of caregivers in accessing mental healthcare services for children with ASD.

**TABLE 1:** Themes and subthemes identified from qualitative interviews with caregivers of children with autism spectrum disorder.

Theme	Subthemes
1. Inadequate access to mental healthcare services	1.1 Psychiatrist distribution and patient population 1.2 Explicit referral pathways 1.3 Accessing allied health services at a primary care level
2. Delayed screening and early diagnosis	2.1 Strengthening nursing staff knowledge through primary care provider training 2.2 Time constraints
3. Limited continuous education to caregivers	3.1 Raising awareness (pamphlets and mass media) 3.2 Managing challenging behaviours.
4. Unmet emotional support and counselling	4.1 Improving coping strategies through support networks and stress management programmes 4.2 Counselling for mental health concerns
5. Inequitable access to specialised care	5.1 Referrals to special needs schools 5.2 Referral to allied health services

### Theme 1: Inadequate access to mental healthcare services

#### Subtheme 1.1: Psychiatrist distribution and patient population

Caregivers reported disparities in access to psychiatric care between the public and private healthcare sectors. Limited availability of psychiatrists and specialised nurses in public facilities led many caregivers to experience prolonged delays in receiving specialised care:

‘They [*doctors at a local hospital*] were quick to diagnose and help him.’ (Participant A)

‘To be honest, I tried public, but it was the waiting and not getting help. When they tell you your child has autism, you want answers, want to see results, you want change, to a lot of questions, but with the public hospital, the process is long. I decided to spend money on private health care, just to get the answers that I need … When we went to the doctors (public hospital), they were fully equipped with knowledge, and they knew what they were doing. They are good. The only challenge is the number of them in the hospital.’ (Participant F)

#### Subtheme 1.2: Explicit referral pathways

While some caregivers experienced smooth referral processes, others encountered repeated visits, delayed responses and initial dismissal of concerns:

‘It took multiple visits and referrals before we finally got to see a psychiatrist who could help.’ (Participant I)

‘The referral from my GP [*general practitioner*] to a specialist was seamless, and I felt supported throughout the process.’ (Participant J)

‘After several visits to the clinic without any progress, I insisted on a referral to a private psychiatrist, and that made all the difference.’ (Participant L)

#### Subtheme 1.3: Accessing allied health services at a primary care level

Some caregivers shared positive experiences with early identification and referrals by clinic staff, while others expressed concern over the effectiveness of group speech therapy sessions because of the individual needs of children on the spectrum:

‘The nurses pointed out the child was delayed for his age, then referred me to a local hospital, where he started seeing an occupational therapist and speech therapist.’ (Participant C)

‘We went to the local clinic, and they referred us to [*redacted-referring hospital*] for occupational therapy.’ (Participant D)

‘So, speech therapy is done as a group. I even ask how that is so? Because this is a spectrum, they cannot be doing therapy together because there are at different levels, that was when they explained to me that these children are a lot [*sic*] and the therapists are few, hence they group them for sessions.’ (Participant H)

### Theme 2: Delayed screening and early diagnosis

#### Subtheme 2.1: Strengthening nursing staff knowledge through primary care provider training

Caregivers found a general lack of ASD knowledge among nursing staff, with developmental delays frequently overlooked during routine clinic visits, and emphasised the need for improved training among primary care providers to enable earlier diagnosis and more informed care:

‘During routine clinic visits, the nurses would ask if he had started walking or talking, but they didn’t recognise any signs of autism.’ (Participant A)

‘He used to cut himself, and he would cry for no reason and walk with his toes.’ (Participant B)

She added that:

‘[*T*]he school had invited the Department of Health … to tell me that there was something wrong with my son when he was in Grade 1 … They didn’t tell me about autism; they just mentioned intellectual disability … A formal diagnosis of ASD was only made at age 16 at Weskoppies Hospital.’ (Participant B)

‘If the doctors had better training on autism, they would have diagnosed my child much earlier.’ (Participant C)

#### Subtheme 2.2: Time constraints

Caregivers often felt rushed during appointments, which limited their ability to discuss their concerns effectively:

‘The doctors seemed overwhelmed and didn’t have enough time to discuss my concerns about my child’s behaviour.’ (Participant D)

‘Every time I went for an appointment, it felt rushed. I never had enough time to explain everything that was wrong with my child.’ (Participant O)

### Theme 3: Limited continuous education for caregivers

#### Subtheme 3.1: Raising awareness (pamphlets and mass media)

Caregivers recommended increased educational outreach, particularly through printed materials, TV programmes and community campaigns:

‘They used to give us pamphlets at [*redacted-referring hospital*] about autism, teaching us not to panic at their behaviour, how to treat them and help them.’ (Participant A)

‘For me, the biggest issue is (lack of) information and posters, and where to seek help. Remember, during the outbreak of HIV, we would have TV shows like Soul City, teaching the communities about HIV and what to do. Why don’t we have programmes about mental health care, maybe say if you suspect this, what is the first step? TV may be too much, and people will be thinking about money, but posters can go a long way, like we had posters from the HIV and the COVID-19 outbreak. Now we have come to understand that mental health is also vital, and we are realising that a lot of issues that we are facing are because people are not in the right mindset. There should be more noise and awareness about that and around how to get help.’ (Participant D)

#### Subtheme 3.2: Managing challenging behaviours

Caregivers appreciated strategies learned through workshops and professional guidance for managing outbursts and meltdowns:

‘The workshops taught me how to manage my child’s meltdowns effectively.’ (Participant F)

‘The occupational therapist provided me with strategies to help my child during difficult moments.’ (Participant G)

‘I wish there were more resources available on how to handle aggressive outbursts.’ (Participant J)

### Theme 4: Unmet emotional support and counselling

#### Subtheme 4.1: Improving coping strategies through support networks and stress management programmes

Caregivers described feelings of loneliness, emotional fatigue and isolation in their caregiving journey, highlighting the importance of support from family and other parents as well as the need for better coping resources and organised support structures:

‘I become emotional when I talk about what it means to be a caregiver of a child with autism. It means I am living a lie; I smile, and I look good, but I’m actually going through a lot, and I am tired. Maybe God saw me fit for this. It’s been 16 years.’ (Participant B)

‘Meeting other parents in similar situations made me feel less alone.’ (Participant D)

‘Only if information about how to take care of children with autism, because it is difficult, you never know what to expect from them.’ (Participant G)

#### Subtheme 4.2: Counselling for mental health concerns

Caregivers reported unmet mental health needs and requested integrated counselling services:

‘We need counselling; I am talking like this because I need that counselling; sometimes I cannot sleep. Last month, I had pain; I had this pain for some time. I went to the clinic, and they gave me tablets; when I went back for a refill, they asked me what was stressing me and what I do not talk about. I told them that I was living with an autistic child, and everyone wanted me to be strong like everyone else … They do not have it, I asked if I could see a psychologist, but they do not have it. I also approached a 24-hour service clinic, and they told me the psychologist who was there had left. I do not even have friends because no one understands what I am going through.’ (Participant B)

‘They [*redacted-public health care centre*] should provide parents with counselling, provide classes, and educate them. Because with my experience, I had to do my own research. But not every parent has access to the knowledge tools. Having a child with autism is not easy, e.g going for family or function gatherings, exposing your child to people with no knowledge of the condition can be challenging, it becomes difficult for both the child and the people, the people may not know how to relate to him. If the child is having a session, perhaps parents should also receive counselling, e.g. dividing the 1-hour session into two parts, 30 minutes for the child and 30 minutes for the caregiver. That would be helpful.’ (Participant F)

### Theme 5: Inequitable access to specialised care

#### Subtheme 5.1: Referrals to special needs schools

Caregivers described challenges enrolling their children in suitable educational settings and stressed the need for clearer referral systems:

‘I wish there were more resources available to help parents navigate the school enrolment process.’ (Participant H)

‘When it comes to looking for a place where you can take your child, where they can learn something, sometimes it becomes very difficult; it depends on the person that you meet there (the Department of Education). The person I met there was not really helpful’ (Participant I)

‘The clinic provided me with information about nearby special schools, which was helpful.’ (Participant N)

#### Subtheme 5.2: Referral to allied health services

Allied health services, such as occupational and speech therapy, were seen as vital but often inaccessible because of high costs, limiting long-term engagement:

‘Yes, occupational therapy, we went there, at [*redacted- referring hospital*]. In speech therapy, they were checking if he could hear or speak. We never went deep with speech therapy or had sessions. Occupational therapy was to teach him how to write and how to do things on his own …’ (Participant B)

‘Those services [*occupational therapy*] help a lot, if the government can assist, because those services are expensive. I noticed that when my child was attending OT, his walking improved. Those services are expensive, and if you do not have money, it’s a problem. After 6 years [*child’s age*], the hospital stops them. For me, I took him for a year because I started late.’ (Participant H)

## Discussion

This study highlights disparities in access to mental healthcare services for children with ASD, aligning with existing research.^17,18^ In low- and middle-income countries, barriers such as limited resources, stigma and under-resourced healthcare systems exacerbate difficulties in obtaining timely and appropriate care for children with ASD.^19^ Global trends indicate that socio-economic status, geographic location and systemic inefficiencies contribute to unequal access to mental healthcare.^19^ Similar barriers have been documented in comparable African settings, including Kenya, Nigeria and Ghana, where caregivers consistently report inadequate health system support, limited specialist availability and high financial burden, underscoring the regional nature of these challenges.^20,21,22^

In South Africa, access to mental healthcare remains unevenly distributed across the private and public healthcare sectors. The medically insured minority can access private healthcare services, where most mental health professionals, including specialists, are concentrated.^23^ In contrast, the public healthcare sector, which serves the majority, faces critical shortages of these specialists.^23^ The scarcity of mental health care professionals at the primary level increases caregiver stress and contradicts the principles of universal health coverage.^23^

Caregivers using public healthcare services in South Africa frequently encounter protracted referral processes, often requiring years of primary healthcare visits before reaching tertiary-level care.^24^ In South Africa, up to 78% of families wait more than a year for access to specialised ASD services.^25^ These delays disproportionately affect low-income families who cannot access private healthcare.^25^ Delays in diagnosis are compounded by the structure of South Africa’s tiered referral system.^14^ Other countries have implemented centralised systems, telemedicine and case managers to address inefficiencies and streamline care and improve access to specialists.^26^ Australia’s integrated model has shown that these strategies can reduce waiting times and improve early diagnosis of neurodevelopmental conditions.^26^

Strengthening primary healthcare interventions through community-based models has proven effective in the early identification of ASD and timely referral for mental health conditions.^27^ Community health workers form the backbone of SA’s primary healthcare outreach, providing home-based support, health education and early identification of developmental needs, which strengthens referral pathways in low-resource communities.^28^ Their role is analogous to SA’s Directly Observed Treatment programme for tuberculosis, where CHWs were central to improving early detection, treatment adherence and outcomes.^29^ This demonstrates how community-embedded models can be leveraged for earlier ASD identification and intervention.

Primary healthcare services are critical entry points for identifying ASD-related conditions. Strengthening the presence of allied health professionals at this level could improve early intervention outcomes. Literature supports both individual and group-based speech therapy for children with ASD, each with unique benefits.^30^ Individual therapy enables highly personalised, child-centred approaches,^30^ while group therapy facilitates peer modelling and social learning.^31^ In public healthcare settings with limited resources, group therapy enables service delivery to more children, but proper child matching and therapist training are essential to ensure optimal outcomes.^31^

Delays in diagnosis are frequently linked to a lack of awareness about ASD among nursing staff and general practitioners. Delays are further aggravated by insufficient ASD-specific knowledge among primary health care providers, contributing to misdiagnosis and late referral.^32^ Notably, a small number of participants reported positive experiences with nursing staff who recognised early signs of ASD, suggesting that individual clinical competence exists within the system but remains inconsistently distributed and unsupported by standardised training protocols.

One practical solution could involve integrating neurodevelopmental screening tools into the Road to Health Card (RTHC), a national health record distributed to all South African children at birth. While currently used to track immunisations, weight and developmental milestones, the RTHC does not currently include screening for neurodevelopmental conditions.^33^ Integrating ASD screening into routine RTHC check-ups could enable earlier detection and referral. This must be accompanied by improved communication and awareness between providers and families.^34^ Local efforts to educate caregivers were praised, and participants advocated for broader awareness campaigns modelled after national human immunodeficiency virus and coronavirus disease 2019 public health responses. Such campaigns are critical in reducing stigma and encouraging early help-seeking behaviour.^35^

Continuing professional development (CPD) in ASD, similar to the compulsory ethics CPD regulated by the Health Professions Council of South Africa, could be mandated for all healthcare providers, with particular emphasis on paediatric nurses and primary care practitioners who serve as the first point of contact for families of children with ASD. In-service training, supported by tertiary institutions, can be scaled up at the primary care level to ensure that providers remain informed about emerging advances in ASD-related research.^36^ Increased awareness of families and knowledge by healthcare professionals may further be enhanced by intersectoral collaboration through various national departments, including health, education and social development.

Currently, South Africa’s public healthcare system is overburdened, with 84% of the population relying on government services, where average diagnostic delays for ASD exceed 3 years.^37^ Specialist shortages, a lack of training among primary care providers, fragmented referral pathways and inconsistent use of screening tools result in only 29.3% of practitioners conducting routine ASD screening at 18–24 months.^37^ The universal health initiatives like the proposed NHI aims to address these disparities through integrated specialist services and improved primary care access.^38^ The findings of this study seems to underscore the urgency of such initiatives, not merely as a financing mechanism, but as a structural remedy for redistributing specialist mental health services to underserved communities and standardising ASD care pathways across all levels of the health system.^38^

In addition to increasing parents’ awareness about ASD, attempts to assist with their stress management and coping strategies may be beneficial. Implementing initiatives such as the Positive Parenting Programme and Autism Speaks’ Caregiver Skills Training, recommended by the World Health Organization,^39^ could empower caregivers in South Africa with practical strategies and reduce caregiving stress. These experiences are consistent with the Double ABC-X model of family stress, which posits that caregiver burden in families of children with ASD is shaped by the interaction between stressors, available resources and coping strategies.^40,41^ This is consistent with local evidence from a South African study, which found that 75% of caregivers of children with ASD experienced some degree of caregiver burden, underscoring the need for improved psychosocial support programmes within the public health sector.^42^

Navigating educational placement remains an important barrier for many families. Participants described delays in school enrolment and difficulties accessing appropriate educational resources. These findings align with global research documenting prolonged waitlists and inconsistent referral practices.^43^

### Limitations

This study has several limitations. Data were collected from a single tertiary hospital in Pretoria, Gauteng, which may limit transferability to other settings in South Africa. As a qualitative study, findings reflect subjective participant experiences and are not intended to be generalisable. The retrospective nature of interviews may have introduced recall bias, and some meaning may have been lost during translation despite verification procedures. Additionally, some demographic and service-related variables were not systematically collected, limiting comparative analysis. Finally, thematic analysis is interpretative and may have been influenced by the principal investigator’s analytical perspective despite reflexivity measures.

### Future study recommendations

The authors recommend future study designs that enable comparative analysis across healthcare sectors and the role of non-governmental organisations in assisting with access to mental health care services.

## Conclusion

This study underscores the need to improve and strengthen access to mental healthcare for children with ASD in South Africa. It further identified key barriers that prompt attention from various stakeholders to improve access to mental healthcare services for children and responsiveness to children with ASD. Enhancing referral efficiency, supporting community-based models and increasing public and provider awareness may contribute to earlier detection, more effective intervention and improved caregiver experiences.
